# The ACCURE-trial: the effect of appendectomy on the clinical course of ulcerative colitis, a randomised international multicenter trial (NTR2883) and the ACCURE-UK trial: a randomised external pilot trial (ISRCTN56523019)

**DOI:** 10.1186/s12893-015-0017-1

**Published:** 2015-03-18

**Authors:** Tjibbe J Gardenbroek, Thomas D Pinkney, Saloomeh Sahami, Dion G Morton, Christianne J Buskens, Cyriel Y Ponsioen, Pieter J Tanis, Mark Löwenberg, Gijs R van den Brink, Ivo AMJ Broeders, Paul HJM Pullens, Tom Seerden, Maarten J Boom, Rosalie C Mallant-Hent, Robert EGJM Pierik, Juda Vecht, Meindert N Sosef, Annick B van Nunen, Bart A van Wagensveld, Pieter CF Stokkers, Michael F Gerhards, Jeroen M Jansen, Yair Acherman, Annekatrien CTM Depla, Guido HH Mannaerts, Rachel West, Tariq Iqbal, Shrikanth Pathmakanthan, Rebecca Howard, Laura Magill, Baljit Singh, Ye Htun Oo, Dmitri Negpodiev, Marcel GW Dijkgraaf, Geert RAM D’Haens, Willem A Bemelman

**Affiliations:** Department of Surgery, Academic Medical Centre, PO Box 22660, 1100 DD Amsterdam, The Netherlands; Department of Surgery, University Hospitals Birmingham, Birmingham, UK; Department of Gastroenterology, Academic Medical Centre, Amsterdam, The Netherlands; Department of Surgery, Meander Medical Center, Amersfoort, The Netherlands; Department of Gastroenterology, Meander Medical Center, Amersfoort, The Netherlands; Department of Gastroenterology, Amphia Hospital, Breda, The Netherlands; Department of Surgery, Flevo Hospital, Almere, The Netherlands; Department of Gastroenterology, Flevo Hospital, Almere, The Netherlands; Department of Surgery, Isala Hospital, Zwolle, The Netherlands; Department of Gastroenterology, Isala Hospital, Zwolle, The Netherlands; Department of Surgery, Atrium Medical Center, Heerlen, The Netherlands; Department of Gastroenterology, Atrium Medical Center, Heerlen, The Netherlands; Department of Surgery, Lucas Andreas Hospital, Amsterdam, The Netherlands; Department of Gastroenterology, Lucas Andreas Hospital, Amsterdam, The Netherlands; Department of Surgery, Onze Lieve Vrouwe Hospital, Amsterdam, The Netherlands; Department of Gastroenterology, Onze Lieve Vrouwe Hospital, Amsterdam, The Netherlands; Department of Surgery, Slotervaart Hospital, Amsterdam, The Netherlands; Department of Gastroenterology, Slotervaart Hospital, Amsterdam, The Netherlands; Department of Surgery, St. Franciscus Hospital, Rotterdam, The Netherlands; Department of Gastroenterology, St. Franciscus Hospital, Rotterdam, The Netherlands; Department of Gastroenterology, University Hospitals Birmingham, Birmingham, UK; Birmingham Clinical Trials Unit, University Hospitals Birmingham, Birmingham, UK; Department of Surgery, University Hospitals Leicester, Leicester, UK; School of Immunity and Infection, University of Birmingham, Birmingham, UK; Clinical Research Unit, Academic Medical Centre, Amsterdam, The Netherlands

**Keywords:** Inflammatory bowel disease, Ulcerative colitis, Appendectomy, Surgery, Disease course

## Abstract

**Background:**

Over the past 20 years evidence has accumulated confirming the immunomodulatory role of the appendix in ulcerative colitis (UC). This led to the idea that appendectomy might alter the clinical course of established UC. The objective of this body of research is to evaluate the short-term and medium-term efficacy of appendectomy to maintain remission in patients with UC, and to establish the acceptability and cost-effectiveness of the intervention compared to standard treatment.

**Methods/Design:**

These paired phase III multicenter prospective randomised studies will include patients over 18 years of age with an established diagnosis of ulcerative colitis and a disease relapse within 12 months prior to randomisation. Patients need to have been medically treated until complete clinical (Mayo score <3) and endoscopic (Mayo score 0 or 1) remission. Patients will then be randomised 1:1 to a control group (maintenance 5-ASA treatment, no appendectomy) or elective laparoscopic appendectomy plus maintenance treatment. The primary outcome measure is the one year cumulative UC relapse rate - defined both clinically and endoscopically as a total Mayo-score ≥5 with endoscopic subscore of 2 or 3. Secondary outcomes that will be assessed include the number of relapses per patient at 12 months, the time to first relapse, health related quality of life and treatment costs, and number of colectomies in each arm.

**Discussion:**

The ACCURE and ACCURE-UK trials will provide evidence on the role and acceptability of appendectomy in the treatment of ulcerative colitis and the effects of appendectomy on the disease course.

**Trial registration:**

NTR2883; ISRCTN56523019

## Background

Ulcerative Colitis (UC) is an inflammatory bowel disease that diffusely affects the mucosa of the colon at variable distance from the anal verge. The aetiology of UC is not fully understood, although it is considered to be multifactorial with genetic and environmental factors leading to an inappropriate immunologic response [1,2]. Cytokine imbalance and the production of inflammatory mediators by activated CD4+ T cells are thought to play an important role in the pathogenesis of UC. T-helper type 2 cells and their cytokines are suggested to enhance the development of UC [1].

The primary treatment of UC is medical, first with 5-aminosalicyclic acids (5-ASA) and/or corticosteroids. More refractory patients need immunosuppression with thiopurines, calcineurin inhibitors or TNF alpha blockers. In disease refractory to medical treatment, a (staged) proctocolectomy with ileo-anal pouch anastomosis is usually performed. Approximately 30% of UC patients eventually require surgery [3-6].

A significant proportion of UC patients will remain on long term medication to maintain remission and prevent relapse, which carries significant morbidity and impacts considerably on quality of life and health resource utilisation. The peak age of onset of UC is 20–35 years old, which means that this condition has a significant impact on working life and potentially procreation and childcare.

Multicentre pooled data suggest that after a flare-up of UC, the annual relapse rate without medication ranges between 40-76%. Even on long-term maintenance therapy up to 40% of patients will still experience at least one relapse within the year, which will require treatment again often including corticosteroids with its incumbent risks and toxicity [7].

Over the past 20 years a substantial body of evidence has accumulated supporting a role for the appendix in the development and course of UC. There is a strong inverse relationship between prior appendectomy (most frequently for appendicitis) and the development of UC, documented through multiple epidemiological and case–control studies from diverse populations [8-10]. Several studies have also investigated the effect of appendectomy on established UC. In a systematic review we have shown that appendectomy might influence the disease course in UC patients, with possible reductions in relapse rates, need for immunosuppression and colectomy rates in UC patients who had an appendectomy, although the heterogeneity of the available studies and subjective nature of the endpoints made direct comparison difficult [11]. Furthermore, it was shown in a T-cell receptor knockout mouse model for colitis that an early appendectomy suppressed inflammation [12]. Another study showed that the proportion of CD4 + CD69+ T cells was significantly increased in the appendix of UC patients, compared to the appendix of controls [13]. In addition, endoscopists have reported on ‘skip lesions’ around the caecal orifice of the appendix in UC patients (referred to as the caecal patch or PARP: peri-appendicular red patch), which is seen even in distal colitis without caecal involvement in 48-86% of patients [10,13-18]. Consequently, the appendix is suggested to be a priming site in the development of UC.

This research body will prospectively evaluate the role of the appendix in UC and the potential effect of appendectomy on the disease course.

## Methods/Design

### Study objective

The objective of these paired studies is to evaluate the short-term and medium-term efficacy of appendectomy to maintain remission in patients with an established diagnosis of mild to moderate ulcerative colitis, to assess the safety and morbidity profile of the intervention, and to explore its acceptability, both to patients with ulcerative colitis and the clinicians responsible for their care.

### Study design

1. ACCURE Trial (Netherlands) - A multicenter randomised clinical trial aiming at patients with an established diagnosis of ulcerative colitis and a disease relapse within 12 months prior to randomisation, medically treated (for treatment with immunomodulators and biologicals a wash out period of 3 months before inclusion is required) until full clinical and endoscopic remission has been achieved as defined by the Mayo score.
2. ACCURE-UK Trial (UK) - This parallel study is a randomised external pilot and feasibility study utilising a matched overall study design, including the same patient population, intervention, and outcome measures.

### Primary and secondary endpoints

The primary endpoint of both trials is the one year cumulative UC relapse rate, defined both clinically and endoscopically as Mayo-score ≥5 with endoscopy sub score of 2 or 3.

Secondary endpoints are the number of relapses per patient in the first year after randomisation, the time to first relapse, disease activity, health related quality of life and treatment costs, and finally the number of colectomies. During longer term follow up beyond one year, disease activity including number of relapses and number of colectomies will be measured.

In addition, ACCURE-UK also aims to explore if the appendectomy intervention is an acceptable treatment option to UC patients and clinicians, establish throughput rates of eligible patients in the UK and estimate the morbidity profile of the intervention in this novel setting.

### Study population

Patients are eligible for both trials when they meet the following inclusion criteria:

1. Aged ≥ 18 years.
2. Histologically confirmed diagnosis of UC.
3. Disease relapse within 12 months prior to randomisation.
4. In remission on 5-ASA or after a wash out period of at least 3 months after treatment with immunomodulators and biologicals (if given).
5. Clinical (Mayo score <3) and endoscopic (Mayo score 0 or 1) remission at time of randomisation.
6. Negative stool culture and C. Difficile toxin.

Patients will be excluded when:

1. A prior appendectomy has been performed.
2. Previous major abdominal surgery that may prevent safe and straightforward appendectomy has been performed.
3. Any suspicion of Crohn’s disease is present.
4. A toxic megacolon or severe ongoing colitis is present at time of randomisation.
5. Diagnosed with active extra-intestinal infections, liver or kidney failure, major lung or heart co-morbidity.

### Participating centres

Nine teaching hospitals in the Netherlands will participate in the ACCURE trial and six hospitals in the United Kingdom in the ACCURE-UK trial, including 4 academic centres.

### Ethics

These studies will be conducted in accordance with the principles of the Declaration of Helsinki and Good Clinical Practice guidelines. Medical ethics approval has been obtained by the medical ethics committee from the Academic Medical Centre in Amsterdam dated April 12th, 2012. Ethical approval has been obtained in the UK via the National Research Ethics System (14/NE/1143; October 24th, 2014). Before randomisation, written informed consent will be obtained from all patients.

### Study outline

**Figure 1:**
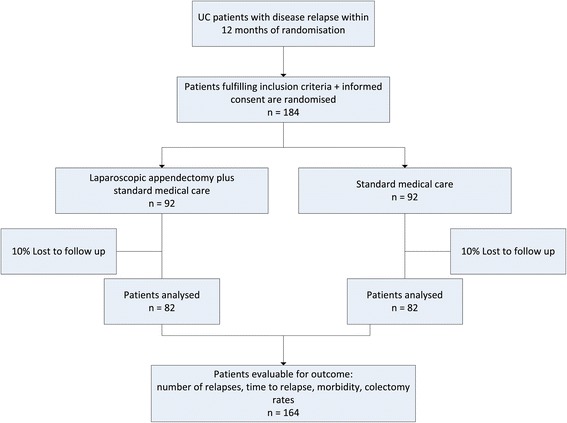
**Study profile.**

Participants will be recruited in the Inflammatory Bowel Disease outpatient clinics of the participating medical centres (Figure 1). Patients eligible for this study are randomised to undergo laparoscopic appendectomy in day care setting within six weeks of randomisation as opposed to no appendectomy. Surgery will be performed in the including centre or one of the coordinating centres; the Academic Medical Centre or University Hospitals Birmingham.

At inclusion, patients will undergo endoscopic mucosal visualisation, either by an ileocolonoscopy or a sigmoidscopy in combination with a faecal calprotectin test that needs to be < 100 ug/g, to confirm remission. At the time of relapse or at the end of the 12 month study period, patients will undergo a full colonoscopy to assess mucosal appearance. The clinical and endoscopic findings will be reported with the Mayo score. Using this 12-point scoring system, disease activity is evaluated based on stool frequency, rectal bleeding, the physician’s global assessment, and endoscopic appearance. In the Mayo score, clinical remission is defined as a Total Mayo score of 2 points or lower, with no individual subscore exceeding 1 point. Mucosal healing is defined as an absolute subscore for endoscopy of 0 or 1.

During the first 12 months of follow up, all patients will receive oral maintenance therapy of 2 grams 5-aminosalicylic acid (Salofalk®, Dr Falk Pharma GmbH, Freiburg, Germany).

Outpatient clinic visits will be performed by the gastroenterologist or research team at 6 weeks and 3, 6, 9 and 12 months after appendectomy or randomisation in patients not undergoing appendectomy; other visits are scheduled on indication. During these contacts the non-invasive 9-point partial Mayo score will be assessed. Patients will complete health related quality of life questionnaires (EQ-5D, EORTC-QLQ-C30-QL and IBDQ) at inclusion and every 3 months thereafter for 12 months. In the 5 years following the study the gastroenterologist will be requested to measure the non-invasive 9-point partial Mayo score every 6 months during outpatient clinic appointments. Three years after randomisation a further colonoscopy will be performed to assess long term mucosal healing in the patients with longstanding remission.

### Laparoscopic appendectomy

Laparoscopic appendectomy is a relatively simple operation that can be done by most surgeons, in day care setting or with a single night stay in hospital. The normal reason to undertake an appendectomy is acute inflammation of the appendix (appendicitis). There is a lifetime incidence risk of 7% and patients diagnosed with this condition almost all undergo appendectomy [19,20]. It is in this forum that the laparoscopic (keyhole) method of removing the appendix has become established over the past 15 years and is now the recommended option owing to its proven faster recovery times and fewer wound complications [20].

The risks of removing a normal or non-inflamed appendix in an elective setting are not known, as published data exclusively refer to patients with confirmed or suspected acute appendicitis undergoing emergency operations by surgeons of variable seniority. It is reasonable to assume that the risk of post-operative complications will be significantly lower in our population than these published rates of 2.5-7.6% from patients with perforated appendicitis [20-22]. Specific complications that have been reported include wound infection, intra-abdominal abscess, iatrogenic bowel perforation, pneumonia and thrombosis. The laparoscopic appendectomy in this study will be carried out under general anaesthesia with the use of 3 trocars or a Single Port system. Laparoscopic appendectomy will be performed by a gastrointestinal or general surgeon with sufficient experience in laparoscopic procedures (>20 procedures/year). When participating centres lack a qualified surgeon the patient will be referred to the Academic Medical Centre, University Hospitals Birmingham or a surgeon that has enough experience in a collaborating hospital nearby.

### Statistical analysis

ACCURE Trial - Group size calculations are based on a clinically relevant reduction in relapse rate from an expected 40% in the control group to 20% in the intervention group. With a 5% two-sided significance level, 82 patients per study arm will be needed to achieve an 80% power to detect such a difference using a Chi-square test. Considering 10% patient drop out we expect to have to include 184 patients in order to analyse 164 patients.

All data-analyses will be performed according to the intention-to-treat principle.

The relapse rate, medication use and time to first relapse of the two groups will be compared with Chi-square testing, Mann Whitney U-testing and survival analysis. Additional mixed-models repeated measures analysis of variance will be used to investigate whether there is a different pattern of change over time between the two study arms in the Mayo score and the EQ-5D, EORTC-QLQ-C30-QL and IBDQ [23].

ACCURE-UK Trial - The target recruitment of this randomised feasibility trial has been set at 48 patients as it was felt this was the minimum number needed to satisfy the objectives. By recruiting 48 patients, 24 will be randomised to the intervention arm. This will provide an estimation of operative morbidity rate and generalisable evidence about the length of stay, time off work and impact upon HRQL. The full cohort (both arms) will provide information on follow-up acceptability and attrition rates. Running the trial at 6 centres across the UK will generate a better understanding of likely patient acceptance rates for the intervention and understand potential barriers to recruitment in different settings.

### Randomisation

In both studies, patient data are entered into a computerized database and following the assignment of an unchangeable computer generated number, patients will be randomised to undergo laparoscopic appendectomy or no appendectomy. Randomisation will be performed stratified by disease localization (rectum, left sided colitis, pancolitis), number of years diagnosed and number of relapses in the past 12 months. Randomisation will take place after all inclusion and exclusion criteria have been verified and informed consent has been obtained.

### Blinding

Blinding of patients and physicians during treatment is unfeasible in this study. Endoscopic procedures will be recorded with photo documentation. The endoscopic follow- up for recurrence will be scored by independent gastroenterologists blinded for the allocated treatment strategy. Pathologists will be blinded to the treatment allocation.

### Data collection and monitoring

An electronic Case Report Form (eCRF) will include general patient data: sex, age, medical history, disease characteristics before and during the study period including Mayo score, appendectomy parameters, complications, mortality, duration of hospital stay and the patients’ responses to the questionnaires. Patients will be followed for a period of 12 months. During this follow-up period patients will complete sets of questionnaires (EQ-5D, EORTC-QLQ-C30-QL and IBDQ). The questionnaires can be filled in online. For this a personal email with the invitation and access code will be sent to the patients by mail. Patients not willing or unable to complete the online questionnaires will receive identical paper questionnaires at their home address, accompanied by a free return envelope.

Patients will be contacted by telephone every 3 months by a study team member (i.e. trial nurse) to assess medication usage, complications, additional interventions, re-admissions, duration of hospital stay and visits to the outpatient clinic, number of days of sick leave and of social in attendance and to ensure completions of the questionnaires.

### Economic evaluation

ACCURE Trial - The economic evaluation of appendectomy for established UC will be performed from a societal perspective as a cost-effectiveness and cost-utility analysis with a time horizon of 12 months. Primary outcomes in these economic evaluations are the costs per patient without relapse and costs per quality adjusted life-year (QALY) respectively. Incremental cost-effectiveness ratios will be calculated as the extra costs per additional patient without relapse or per QALY gained. Furthermore, the cost-effectiveness per prevented relapse will be calculated. Additional sensitivity analyses will determine how changing treatment costs might impact the results.

Direct medical costs and indirect non-medical costs arising from losses in productivity will be assessed. National unit costs will be used for the various health care components, complemented by results from activity based costing when needed. Both, the human capital approach as well as the friction cost method will be applied to the indirect costs of sick leave from work.

The EQ-5D scoring profiles at successive measurements during follow-up will be used to derive the corresponding health utilities. This is done by applying computer algorithms available from the literature. These algorithms reflect preferences in the general population for various health states and have been determined using time trade-off elicitation techniques [24,25]. Having determined the health utilities, a QALY estimate for each patient can be calculated, accounting for the lengths of the periods in-between successive measurements.

The ACCURE-UK trial does not contain any planned health economic assessment as this does not form part of the feasibility aims.

### Histopathological evaluation

Samples of appendicular tissues will be collected and investigated at the Tytgat Laboratory of the Academic Medical Centre Amsterdam according to a standard operating procedure. Resected appendices will be analysed by immunohistochemistry, FACS analysis and microbiota analysis will be performed. In the UK, translational research samples will be centralised to the University of Birmingham for analysis.

### Patient safety

An independent Data and Safety Monitoring Committee (DSMC) has been established to assure proper data safety and relevance monitoring. The committee will guard the safety of the included patients, give advice on continuation of the study upon superiority of one of the types of treatment, and will guard the methodological quality of the study. An interim review will be performed at 25, 50 and 100 included patients. At 6 weeks after inclusion of these patients the trial’s safety data will be evaluated. According to the Good Clinical Practice guidelines, a list of Serious Adverse events is defined. All events on this list have to be reported by the local investigators to the trial coordinators within 24 hours after the event. The DSMC will be supplied with the number of (serious) adverse events in both groups at the three mentioned time points. If there is a skewed distribution of the number of (serious) adverse events between the two groups an efficacy analysis can be performed at the discretion of the DSMC.

### Inclusion period

The ACCURE Trial (Netherlands) included the first patient in September 2012, the ACCURE-UK Trial commenced inclusion in December 2014. Full inclusion is expected in August 2016.

## Discussion

Chronic relapsing diseases such as ulcerative colitis (UC) incur a considerable long-term health burden to the patients and health care systems. Early interventions that reduce the rate of relapse and colectomy would offer considerable benefit. Many patients with UC live with a substantial symptom burden despite medical treatment; three quarters report that symptoms affect their ability to enjoy leisure activities and two thirds of patients feel that symptoms affect their ability to perform at work [26]. Therefore, it is imperative to explore novel treatment options.

As early as 1987 the appendix was suggested to play a role in ulcerative colitis, when an epidemiological study found a lower appendectomy rate in UC patients compared to healthy controls [27]. In the following years the involvement of the appendix in UC was confirmed by various other studies, suggesting the appendix’ role in the immunologic cascade in the colonic mucosa of UC patients [10,13-18,28]. The available literature suggests a preferable clinical course in appendectomised patients with UC, but strong evidence is lacking [11].

The primary outcome of interest in this research is the proportion of patients with UC relapse, as defined by the Mayo score. All patients in the study will be treated with 2 grams 5-ASA maintenance therapy. With this maintenance therapy, approximately 40% of the patients with UC will have a disease relapse within one year [7,29].

If an appendectomy can protect UC patients from future use of expensive or potentially hazardous medication or even surgery, the initial additional costs and potential side effects of appendectomy will be offset by substantial gain in health and reduction in costs later on. This is especially true for appendectomy, as this is a relatively simple procedure that can be performed in day care.

The ACCURE trial is a pragmatic multicentre trial which is appropriately powered to detect any therapeutic benefit from appendectomy in UC sufferers. If the ACCURE-UK randomised feasibility trial shows that the intervention appears to be acceptable to clinicians and patients in the UK, and sufficient patients can be recruited and followed-up, we anticipate undertaking further parallel major multicentre phase III trial of the intervention in the UK in the next year or two. Together, these two trials will provide a powerful evidence base of the clinical efficacy of the intervention in a diverse cohort.

Appendectomy is not currently employed as a therapeutic treatment for UC anywhere in the world. If our research shows it to be safe, efficacious and cost-effective, widespread uptake would be anticipated.

## Abbreviations

ACCURE: The effect of Appendectomy on the Clinical Course of UlceRatiVe colitis
UC: Ulcerative colitis
5-ASA: 5-aminosalicyclic acids
PARP: Peri-appendicular red patch
EQ-5D: Euroqol 5 dimensions
EORTC-QLQ-C30-QL: European Organization for Research and Treatment of Cancer quality of life
IBDQ: Inflammatory bowel disease questionnaire
UK: United Kingdom
