# Erratum to: The ACCURE-trial: the effect of appendectomy on the clinical course of ulcerative colitis, a randomised international multicenter trial (NTR2883) and the ACCURE-UK trial: a randomised external pilot trial (ISRCTN56523019)

**DOI:** 10.1186/s12893-015-0113-2

**Published:** 2016-01-04

**Authors:** Tjibbe J. Gardenbroek, Thomas D. Pinkney, Saloomeh Sahami, Dion G. Morton, Christianne J. Buskens, Cyriel Y. Ponsioen, Pieter J. Tanis, Mark Löwenberg, Gijs R. van den Brink, Ivo A. M. J. Broeders, Hendrikus J. M. Pullens, Tom Seerden, Maarten J. Boom, Rosalie C. Mallant-Hent, Robert E. G. J. M. Pierik, Juda Vecht, Meindert N. Sosef, Annick B. van Nunen, Bart A. van Wagensveld, Pieter C. F. Stokkers, Michael F. Gerhards, Jeroen M. Jansen, Yair Acherman, Annekatrien C. T. M. Depla, Guido H. H. Mannaerts, Rachel West, Tariq Iqbal, Shrikanth Pathmakanthan, Rebecca Howard, Laura Magill, Baljit Singh, Ye H. Oo, Dmitri Negpodiev, Marcel G. W. Dijkgraaf, Geert R. A. M. D’Haens, Willem A. Bemelman

**Affiliations:** Department of Surgery, Academic Medical Centre, Amsterdam, 1100 DD The Netherlands; Department of Surgery, University Hospitals Birmingham, Birmingham, UK; Department of Gastroenterology, Academic Medical Centre, Amsterdam, The Netherlands; Department of Surgery, Meander Medical Center, Amersfoort, The Netherlands; Department of Gastroenterology, Meander Medical Center, Amersfoort, The Netherlands; Department of Gastroenterology, Amphia Hospital, Breda, The Netherlands; Department of Surgery, Flevo Hospital, Almere, The Netherlands; Department of Gastroenterology, Flevo Hospital, Almere, The Netherlands; Department of Surgery, Isala Hospital, Zwolle, The Netherlands; Department of Gastroenterology, Isala Hospital, Zwolle, The Netherlands; Department of Surgery, Atrium Medical Center, Heerlen, The Netherlands; Department of Gastroenterology, Atrium Medical Center, Heerlen, The Netherlands; Department of Surgery, Lucas Andreas Hospital, Amsterdam, The Netherlands; Department of Gastroenterology, Lucas Andreas Hospital, Amsterdam, The Netherlands; Department of Surgery, Onze Lieve Vrouwe Hospital, Amsterdam, The Netherlands; Department of Gastroenterology, Onze Lieve Vrouwe Hospital, Amsterdam, The Netherlands; Department of Surgery, Slotervaart Hospital, Amsterdam, The Netherlands; Department of Gastroenterology, Slotervaart Hospital, Amsterdam, The Netherlands; Department of Surgery, St. Franciscus Hospital, Rotterdam, The Netherlands; Department of Gastroenterology, St. Franciscus Hospital, Rotterdam, The Netherlands; Department of Gastroenterology, University Hospitals Birmingham, Birmingham, UK; Birmingham Clinical Trials Unit, University Hospitals Birmingham, Birmingham, UK; Department of Surgery, University Hospitals Leicester, Leicester, UK; School of Immunity and Infection, University of Birmingham, Birmingham, UK; Clinical Research Unit, Academic Medical Centre, Amsterdam, The Netherlands

## Erratum

Following publication of the original version of this article in *BMC Surgery* [1] it was brought to our attention that the names of two authors were spelled incorrectly. The name of author Ye H Oo was incorrectly spelled as ‘Ye Htun Oo’ and the name of Hendrikus J.M. Pullens was incorrectly spelled as ‘Paul HJM Pullens’. We apologize for the inconvenience this may have caused.
